# Climate change control: the Lindahl solution

**DOI:** 10.1007/s11027-017-9758-8

**Published:** 2017-08-19

**Authors:** Loek Groot, Julia Swart

**Affiliations:** 1 Utrecht University School of Economics, P.O. Box 80125, 3508 TC Utrecht, The Netherlands; 2 Utrecht University School of Economics, P.O. Box 80125, 3508 TC Utrecht, The Netherlands

**Keywords:** Nash, Lindahl, Tradable permits, Equity, Efficiency, Burden sharing rule, D610, D63, H410, Q01, Q5

## Abstract

The main purpose of this paper is to evaluate different burden sharing rules with respect to abatement of carbon emissions. We evaluate seven different rules both in terms of their redistributive impact and by the extent to which they realize the aim of optimal abatement. We show that the Lindahl solution, where the burden sharing rule of carbon abatement is determined by each region’s willingness to pay, is to be preferred above the non-cooperative Nash outcome. Poor regions however would prefer the social planner outcome with a global permit market, because then the burden sharing rule has a secondary role of income redistribution by means of transfers from rich to poor, on top of its primary role of assigning abatement burdens. Based on these findings, we argue that in order to control global greenhouse gas emissions, the level of individual country emission abatement effort should be a function of their willingness to pay to curb climate change, rather than their historical emissions or ability to abate.

## Introduction

Article 2 of the 2015 United Nations Climate Change Conference Paris Agreement aims at limiting global warming to well below 2 °C and “Making finance flows consistent with a pathway towards low greenhouse gas emissions and climate-resilient development.”^1^ Contrary to the Kyoto protocol, there is no detailed country- and time-specific path to reach these goals, but instead a bottom-up approach where each country can set its own “nationally determined contributions” to cut greenhouse gas (GHG) emissions, without an enforcement mechanism. Apparently, the specifics of burden sharing is relegated to the 5-yearly periodical global stocktake specified in Article 14, starting in 2023, although Article 4 states that developed countries should make absolute reductions, against for the near future just mitigation efforts for developing countries and of mobilizing USD $100 billion per year to fund climate policies financed by the developed countries.

Long before Paris 2015, there has been an ongoing and flourishing debate among scientists about the time path to contain global warming. The timing issue is largely concerned with the choice of appropriate discount rates (Weitzman 2001; Nordhaus 2007; Heath 2013) and how to assess the risk of unlikely but potentially disastrous outcomes, e.g., reversal of the “thermohaline circulation,” also known as the “Great Ocean Conveyor Belt” of the Gulf Stream and the exacerbated release of methane due to global warming from the Arctic permafrost. Here, we abstract from the time path and concentrate on how the abatement burdens should be allocated across regions in the world at a given point in time. Given the urgency to reduce GHG emissions worldwide, the most difficult issue is how the burdens are distributed across regions. Since climate change is a global public good, it requires a global burden sharing rule. The two dominant guiding principles for fair burden sharing are the polluter pays principle and the ability to pay principle. For instance, the Stern Review (Stern 2007, p. 23) states that based on income, historic responsibility, and per capita emissions, rich countries should take the primary responsibility to combat climate change. However, the literature on burden sharing emphasizes the free-riding problem as a consequence of climate change being a public good (Gupta 1997). Thus, one important strand in the literature has analyzed the challenge of relating damage caused by climate change to economic activities, which are often occurring in distinct places (see, for example, Edwards and Miller 2001; Voigt 2008 and Dellink et al. 2009). This paper contributes to this literature by avoiding the problem of having to establish the causation from polluter to damage experienced, by turning instead to countries own interest. In this paper, we derive the burden sharing rule following the Lindahl equilibrium (hereafter LE), where burden shares are not so much determined by ability to pay or (historical) emissions, but by countries’ interest, e.g., due to expected damages, to combat global warming.

To illustrate the LE by means of a simple example, suppose two persons share a household in which the cleanliness of the house is considered a public good. The problem of burden sharing is how many hours every member of the household has to spend on cleaning. A simple 50–50 split will not do, because one person may prefer to have the kitchen or closet much cleaner than the other, so even at first glance, fair 50–50 split will not solve the question how many hours in total will have to be spent on cleaning, that is, the provision level of the public good. The LE will identify a unique level of the public good with shares assigned to each household member in such a way, that given the assigned shares, each member will choose the same (Lindahl) level of public good provision with the shares summing up to unity. However, since shares are proportional to marginal willingness to pay (WTP), household members have an incentive not to reveal their true preferences in order to easy- or free-ride on the efforts of the other.

Although one may question how plausible it is that people durably living in a household are able and willing to hide true preferences to get an advantage in burden sharing at the expense of the other member, with many agents sharing a public good strategic misrepresentation of preferences can indeed be a serious problem. With respect to an agreement involving many states, the identified LE from the scientist’ drawing table is therefore only useful as long as the analysis is based on easily observable variables such as GDP, population size, GDP per capita, energy consumption, GHG emissions, and to be expected damages from climate change that are not easy to manipulate.

In the burden sharing debate of climate change, surprisingly little attention is paid to the LE.^2^ In the LE, each country is assigned an abatement share in such a way that given their assigned share, they all want the same level of the global public good—in this case global carbon emission abatement (for the derivation of Lindahl equilibria in public goods models, see Sandler and Murdoch 1990; Mas-Colell 1989; Shitovits and Spiegel 1998, 2003). In such a LE, each country contributes to the global provision level according to its WTP. The saliency of WTP is that any International Environmental Agreement in which some countries have to contribute less than their WTP is a pity, because they are prepared to abate more. At the same time, a country will be reluctant to contribute in excess of its WTP, for instance, if it is prescribed to do so by invoking the polluter pays’ principle or ability to pay principle. Therefore, any deviation from the LE would mean that either some countries contribute less than their actual WTP, which is undesirable if we are to realize the aim of mitigating GHG emissions, or that some countries are supposed to contribute more than they are willing to, making the agreement unstable. Therefore, it is important to assess the burden sharing rule according to the LE and to check how it fares compared to other burden sharing rules.

Buchholz and Peters (2007, 2008) have identified the main fairness properties of the LE. They show not only that the LE is efficient (i.e., satisfying the Samuelson condition for the optimal supply of the public good) but also that the benefit principle (described as “everyone pays what he gets”) and the axiom of proportional contributions (meaning that cost shares are proportional to marginal WTP) are satisfied. Despite these attractive properties, the neglect of the LE should not come as a surprise, since in the economic literature on burden sharing to provide a public good, the LE is said to be merely of theoretical interest, mainly for two reasons: first, the difficulty to assess objectively for each country its WTP and second, the incentive to strategically misrepresent preferences. The best illustration of the practical insignificance of the LE in the debate on climate change is that as far as we know, there is no study with an empirical simulation of the abatement burdens across countries or regions in a LE, a lacuna we hope to fill in this paper.

The structure of this paper is as follows. Section 2 presents the sub-optimal non-cooperating Nash model. Section 3 presents the model with a social planner but without the instrument of redistribution, which, together with the Nash model, are used as benchmarks for the LE. Section 4 identifies the LE in the global burden sharing of abatement. The empirical part is presented in Section 5. In the first simulation, the world is divided into two blocks of rich (Annex I) and poor (Annex II) countries; in the second, five regions are distinguished. The different burden sharing rules are evaluated by two criteria, the extent to which global abatement is optimal and the required degree of redistribution by means of transfers from rich to poor countries. The final section summaries and concludes.

## Non-cooperative Nash with and without permit markets

In modeling climate change and abatement, many choices have to be made. Does one take a static or a dynamic perspective, is the externality arising in consumption or production or both, is the approach rooted in welfare economics or game theory, what is the appropriate discount rate, and so on. In general, many of the choices made here are motivated by keeping the model as simple as possible in order to derive burden sharing rules of abatement under different regimes. In the first regime, serving as a benchmark for the models presented in subsequent sections, countries are assumed to follow their self-interest in a non-cooperative way. Following the withdrawal of the USA on 1 June 2017 from the Paris agreement, ratified on 5 October 2016 and due to the absence of a burden sharing rule and enforcement mechanism, it is not exaggerated to claim that the Nash model, where each country pursues its own interest taking into account the behavior of others, might still be an appropriate workhorse carrying a sense of realism in case the bottom-up Paris approach fails. Cramton and Stoft (2010) go so far to say that after Kyoto, “In fact there is no clear evidence that we have done even as well as the public-goods Nash equilibrium.” First, we model non-cooperative behavior without and with a permit market.

### Non-cooperative Nash without a permit market

In the model, utility (*u*_*i*_) is a function of per capita income available for consumption (${y}_i^c$) and the level of worldwide abatement (*A*), the former being a private good and the latter a global public good. The chosen abatement level in country *i* under Nash behavior (*A*_*i*_), taking the abatement effort of others as given (*A*__*i*_), can be thought of as its emission under a business-as-usual (BAU) scenario minus its actual emission. Country *i* with population *P*_*i*_ is endowed with resources *R*_*i*_, which can be devoted to either consumption (${P}_i{y}_i^c$) or to finance abatement costs (*C*_*i*_(*A*_*i*_)). The endowment *R*_*i*_ can be interpreted as GDP without any cost of abatement, in which case per capita income for consumption equals resources per capita. The Lagrange function for country *i* can be stated as

1 $$ L\left({y}_i^c,{A}_i\right)={P}_i{u}_i\left({y}_i^c,{A}_i+A{\_}_i\right)+{\lambda}_i\left[{R}_i-{P}_i{y}_i^c-{C}_i\left({A}_i\right)\right] $$

with the first term on the right-hand side (RHS) the objective and the second term the resource constraint and *λ*_*i*_ the Lagrange multiplier. Differentiating with respect to per capita consumption and abatement, where variables in subscripts denote derivatives, gives as first-order conditions:

2a $$ {P}_i{u}_{y_i^c}={\lambda}_i\kern0.5em {P}_i\Rightarrow {u}_{y_i^c}={\lambda}_i $$

2b $$ {P}_i{u}_{i,A}={\lambda}_i{C}_{A_i} $$

These two optimum conditions can be summarized as

2c $$ {P}_i\frac{u_{i,A}}{u_{y_i^c}}={C}_{A_i}^N\kern0.5em \Rightarrow {MSB}_{i,A}={C}_{A_i}^N{MU}_{y_i^c} $$

Equation 2c states that the Samuelson rule for the optimal provision of the public good—the sum (*P*_*i*_) of the marginal rate of substitution (${u}_{i,A}/{u}_{y_i^c}$) between abatement and per capita consumption must be equal to the marginal cost of abatement (${C}_{A_i}^N$)—is only applied at the national level. Expressed differently, each country only abates up to the point at which their national marginal social benefits (MSB) are equal to the marginal cost of abatement times the marginal utility of per capita income (MU). The sub-optimality arising under Nash is twofold. First, the positive externalities of abatement in one country for the rest of the world are not taken into account. Second, marginal abatement costs differ between countries, so total abatement is not produced against minimum cost (as shown by Chichilnisky and Heal 1994; Sandmo 2003, 2007; Eyckmans et al. 1993; Sheeran 2006). Overall, abatement levels will be too low and the marginal cost of abatement (hence the abatement level) in a country will be higher the larger its population size and the lower its marginal utility of per capita income. Note that it is assumed that abatement is decided on the national level. If governments would not be in charge to (negotiate and) impose domestic abatement levels, then we would have an atomistic world and under Nash everyone would only mitigate its contribution to global warming up to the point where the private marginal benefits equals private marginal costs.^3^

### Non-cooperative Nash with a permit market

The second sub-optimality can be removed by adopting a worldwide cap-and-trade system—so for each country, the optimal actual abatement (*A*_*i*_) will be determined by where their marginal cost of abatement equals the global permit price (*q*)—while at the same time allowing countries to choose their own target abatement levels (*T*_*i*_). To see how this works out, the resource constraint changes into

3 $$ {R}_i={P}_i{y}_i^c+{C}_i\left({A}_i\right)+q\left({T}_i-{A}_i\right) $$

According to Eq. 3, if the actual abatement in a country is lower than its chosen target level of abatement, then it has to buy additional emission permits against a uniform permit price of *q*. Substituting the world abatement constraint *A* = *T*_*i*_ + *T*__*i*_ in the utility function and including the new resource constraint of Eq. 3 in the Lagrange function gives

4 $$ L\left({y}_i^c,{A}_i,{T}_i\right)={P}_i{u}_i\left({y}_i^c,{T}_i+T{\_}_i\right)+{\lambda}_i\left[{R}_i-{P}_i{y}_i^c-{C}_i\left({A}_i\right)-q\left({T}_i-{A}_i\right)\right] $$

Differentiation with respect to per capita income and abatement gives

5a $$ {u}_{y_i^c}={\lambda}_i $$

5b $$ -{\lambda}_i\left[{C}_{A_i}-q\right]=0\Rightarrow {C}_{A_i}=q $$

For total abatement, which will equal the global sum of the national target abatement levels, the chosen target levels are crucial. Each country will choose its target level according to

5c $$ \frac{\partial L\left({y}_i^c,{A}_i,{T}_i\right)}{\partial {T}_i}={P}_i{u}_A\frac{\partial A}{\partial {T}_i}-{\lambda}_i\left[{C}_{A_i}\frac{\partial {A}_i}{\partial {T}_i}+\frac{\partial q}{\partial {T}_i}\left({T}_i-{A}_i\right)+q\left(1-\frac{\partial {A}_i}{\partial {T}_i}\right)\right]=0 $$

Using the optimum condition of Eq. 5a, dividing by ${u}_{y_i^c}$ and since ∂*A*/∂*T*_*i*_ = 1,^4^ the optimum condition of Eq. 5c can be rewritten as

6 $$ {P}_i\frac{u_A}{u_{y_i^c}}=\frac{\partial {A}_i}{\partial {T}_i}\left({C}_{A_i}-q\right)+q+\frac{\partial q}{\partial {T}_i}\left({T}_i-{A}_i\right) $$

Because of the global permit market, marginal cost will be equalized everywhere to the permit price *q* (see Eq. 5b), so the first term in brackets will be zero and Eq. 6 reduces to

7 $$ {P}_i\frac{u_A}{u_{y_i^c}}=q+{q}_{T_i}\left({T}_i-{A}_i\right) $$

Equation 7 expresses that in choosing the optimal target level, each country equates its marginal social benefit (LHS) to the permit price plus the effect of a higher chosen target level on the permit price (${q}_{T_i}$) times the volume of permits bought or sold by country *i* (the same result is obtained by Cramton and Stoft 2010). In a global permit market ∂*q*/∂*T*_*i*_ = ∂*q*/∂*T*_*j*_ = ∂*q*/∂*T* = *q*_*T*_ and summing both sides of Eq. 7 over all countries results in

8 $$ {\sum}_{i=1}^n{P}_i\frac{u_A}{u_{y_i^c}}= nq+{q}_T{\sum}_{i=1}^n\left({T}_i-{A}_i\right) $$

By definition, the last term is zero when the permit market clears, so Eq. 8 boils down to the global sum of marginal benefits of abatement (the LHS) to be equal to the permit price times the number of countries (the RHS). Although the second sub-optimality of the non-cooperating Nash solution without a global permit market is removed now that the same good abatement is produced at uniform instead of differentiated marginal costs, the first sub-optimality is still there; the price of abatement is, from a world point of view, much too low^5^ because the LHS of Eq. 7 does not contain the global but only the national marginal benefits of abatement.

## A social planner without and with a permit market

Now, suppose countries agree to install a social planner (labeled S) to redress the sub-optimalities of the Nash outcome. If S is given not only the power to set the burden sharing rule for abatement but also the power to redistribute income, the global welfare maximizing outcome will be equality of marginal utilities of income across countries and uniform marginal cost of abatement to ensure production efficiency (see Appendix 1). Although equity and efficiency are achieved simultaneously, it is not realistic to assume that in order to solve the global warming problem, however serious it may be, sovereign rich countries are prepared to equalize their per capita incomes to that of the rest of the world. Therefore, a more realistic version of S is that it lacks the instrument of income redistribution but is still given the restricted mandate to devise an optimal burden sharing abatement rule. We will see that the optimal rule requires the marginal welfare cost of abatement to be equal across countries. We distinguish S without and with the power to install a global permit market.

### A social planner without a permit market

A distinction can be made whether or not S has to operate under an external global abatement constraint. Suppose that all countries agreed that a required level of global abatement (*A* ^∘^), e.g., stipulated by the IPCC relative to BAU emissions, has to be met. This will add a pollution constraint to the exercise, and the only decision by S is to assign the abatement burdens *A*_*i*_ such that their sum equal *A* ^∘^. Thus, S maximizes welfare over all countries subject to the global abatement constraint and all national resource constraints:

9 $$ L\left({y}_i^c,{A}_i,{A}^{\circ}\right)={\sum}_{i=1}^n{P}_i{u}_i\left({y}_i^c,{A}^{\circ}\right)+\mu \left[{\sum}_i{A}_i-{A}^{\circ}\right]+{\sum}_i{\lambda}_i\left[{R}_i-{P}_i{y}_i^c-{C}_i\left({A}_i\right)\right] $$

Note that the only difference of this Lagrange function with the one of S with the power to make cross country lump sum income transfers (see Appendix 1) is that for the former, there is a resource constraint for each country *i* (see the last term in Eq. 9), instead of just one world resource constraint ($\lambda {\sum}_i\left[{R}_i-{P}_i{y}_i^c-{C}_i\left({A}_i\right)\right]$) under a social planner with income redistributive powers. Differentiating Eq. 9 with respect to ${y}_i^c$, *A*_*i*_ and *A* ^∘^ give

10a $$ {u}_{y_i^c}={\lambda}_i $$

10b $$ \mu ={\lambda}_i{C}_{A_i} $$

10c $$ {\sum}_i{P}_i{u}_A=\mu $$

According to Eq. 10a, marginal utility of per capita income is country specific. Because there is no income redistribution, marginal utility of per capita income in poor countries will be higher than in rich countries,^6^ and therefore, the marginal cost of abatement will be set lower in poor countries (see Eq. 10b). Note that if no external global abatement restraint is imposed, S will maximize Eq. 9 also with respect to global abatement, ensuring the optimal level of total abatement as specified by Eq. 10c. The external global abatement level *A* ^∘^ may have been set too high, too low, or just right, and only in the latter case is the shadow cost of global abatement (*μ*) equal to the global sum of marginal abatement benefits (∑_*i*_ *P*_*i*_ *u*_*A*_), as specified by Eq. 10c. Thus, only if the global abatement level is set at the right level, the optimum conditions of Eqs. 10a–c can be summarized as the following Samuelson rule:

11 $$ \frac{\sum_{j=1}^n{P}_j{u}_A^j}{u_{y_i^c}}={C}_A^{i,S}\kern0.5em \Rightarrow {MSB}_w={MC}_A^{i,S}{MU}_y^i. $$

The numerator in the LHS of Eq. 11, the global sum of marginal benefits of abatement (*MSB*_*w*_), is a world total and so not country specific. The denominator, marginal utility of per capita income, is country specific. As a consequence, marginal cost of abatement (the RHS) is also country specific. As the alternative expression of Eq. 11 shows, S distributes the burden of abatement in such a way that, for each country, its marginal cost of abatement times the marginal utility per unit of income—this product can be interpreted as the marginal welfare cost of abatement—is equalized to the global marginal benefits of abatement. All other things equal, poor countries, having a high marginal utility of per capita income, will be assigned a low abatement level so that their (marginal) cost of abatement will be low. Summarizing, in comparison to the non-cooperative case, S is guided by two rules in the maximization of world welfare. First, by allocating abatement burdens to individual countries, the global abatement benefits are relevant, not the national. Second, instead of national marginal abatement cost equated to (marginal) benefits of abatement, now the marginal welfare cost per unit of abatement is equalized across countries.

Comparing Eqs. 11 and 2c shows that the first Nash sub-optimality of not taking positive externalities of abatement in one country to the rest of the world into account is now removed, but the second sub-optimality of differentiated, country-specific, marginal cost of abatement is still present due to the absence of a global permit market. This sub-optimality can be removed by empowering S to install a global permit market.

### A social planner with a global permit market

To remove the second sub-optimality of non-uniform marginal cost of abatement, assume that countries allow the social planner to implement a global permit market (labeled as SP),^7^ which solves the production inefficiency of abatement produced in different countries against different marginal costs. As before, actual abatement levels in each country will be uniquely determined by the equality of marginal cost of abatement (${C}_{A_i}$) and the global permit price (*q*), irrespective of the particular choice by SP of the burden sharing rule *T*_*i*_. The global permit price will either be determined by the chosen level of global abatement *A* ^∘^ by the IPCC or by the planner’s optimal choice of the global abatement level, again irrespective of the sharing rule *T*_*i*_. Hence, if the SP can operate without any constraint in setting *T*_*i*_, to maximize global welfare, the planner will choose *T*_*i*_ in such a way that given the optimal domestic abatement levels determined by where marginal abatement costs equals the global permit price, the resulting transfer payments *q*(*T*_*i*_ − *A*_*i*_) will equalize marginal utility of per capita incomes, implying uniform per capita income as under the lump sum social planner with unconstrained power to redistribute.^8^ Instead of lump sum redistribution, the same redistribution is established by transfer payments following the chosen target abatement levels.^9^

Therefore, a more constrained mandate for SP has to be adopted. For practical reasons (see also the empirical section), we chose to constrain SP in such a way that for each country or region, the target abatement level is set equal to the actual abatement level under S. As a consequence, under SP, the same global abatement level as under planner S without permit market results. The advantage for the rich countries is that their cost will decline, since the global permit price is below their marginal cost of abatement without emission trading. Poor countries will benefit because their target levels are pitched at the low abatement levels stipulated by Eq. 11 and they become consequently sellers of permits on the permit market. Switching to a permit market requires that at the country level, the consumption constraint is adjusted to include its dealings on the permit market, so each country now faces the Lagrangian:

12 $$ L\left({y}_i^c,{A}_i\right)={P}_i{u}_i\left({y}_i^c,A\right)+{\lambda}_i\left[{R}_i-{P}_i{y}_i^c+{C}_i\left({A}_i\right)-q\left({T}_i-{A}_i\right)\right] $$

Since the SP is given the authority to set the burden sharing rule *T*_*i*_, each country takes its assigned burden *T*_*i*_ as given, which gives first-order conditions:

13a $$ {u}_{y_i^c}={\lambda}_i $$

13b $$ {C}_{A_i}=q $$

Thus, although SP is constrained in setting the target-level abatements at the actual abatement levels if there would be no permit market, so ${T}_i^{SP}={A}_i^S$, the global permit market incentivizes individual countries to abate up to the point where their marginal cost are equal to the permit price. Both inefficiencies of the non-cooperative Nash outcome are then removed. Because of the permit market, the production inefficiency is removed and the IPCC or SP can impose the required global abatement level *A* ^∘^.

## The Lindahl solution

From a moral point of view, the acceptability of the Lindahl solution is hampered because it is in conflict with both the polluter pays principle and ability to pay principle. Gardiner (2004, p. 590) concludes that: “… there is a great deal of convergence on the issue of who has primary responsibility to act on climate change. The most defensible accounts of fairness and climate change suggest that the rich countries should bear the brunt, and perhaps even the entirety, of the costs.” In the same vein, the Executive Summary of the Stern Review (Stern 2007, p. 23) states that “Securing broad-based and sustained co-operation requires an equitable distribution of effort across both developed and developing countries. There is no single formula that captures all dimensions of equity, but calculations based on income, historic responsibility and per capita emissions all point to rich countries taking responsibility for emissions reductions of 60–80% from 1990 levels by 2050.” Apparently, both Gardiner and Stern favor a burden sharing rule in which the rich countries bear the lion share of the costs due to their higher ability to pay and to the polluter pays principle.

However, assessing burdens to combat climate change is not merely a morality play. Countries are sovereign and a burden sharing rule based on moral principles—such as the polluter pays principle or the ability to pay principle—is only as strong as the commitment of countries to these principles.^10^ The ability to pay or paying as polluter may not be in line with a countries’ willingness to pay.^11^ Our proposed burden sharing rule is based on countries’ willingness to contribute to combat climate change, which naturally leads to the Lindahl solution to the optimal public good provision level. The Lindahl solution has two major disadvantages. Firstly, the willingness to pay is not only influenced by the expected damage but also by the adaptation costs, which puts many poor countries in a precarious position if they lack the resources and know-how for adaptation. Secondly, contributions according to willingness to pay may be squarely at odds with contributions based either on the ability to pay and the polluter pays principle. For the sake of argument, suppose that the USA is protected from any consequences of climate change and that only the rest of the world would suffer damages. According to ability to pay and the polluter pays principle, the USA would have to contribute heavily, but its willingness to pay might be low. Analogous, the burden assigned to a poor country at sea level according to ability to pay and polluter pays will be low, but relatively high according to willingness to pay. In theory, countries that would benefit from global warming (e.g., Russia, Canada, and Greenland) may have a negative willingness to pay, which implies that they have to be compensated for their participation in an international agreement to abate greenhouse gases. This suggests that concerns of redistribution between rich and poor should be excluded in considerations of how to assess fair burden sharing to combat climate change. What we propose is therefore that climate change policy must be evaluated on its own merits, and the same goes for policies to address global income inequality (see, e.g., Milanovic 2016).

### Lindahl solution without permit market

In the literature, the LE is mostly interpreted in terms of cost shares, but here instead, each country’s share is defined relative to the total abatement level,^12^ so willingness to pay has to be interpreted as willingness to abate or contribute. As before, we make a distinction between the Lindahl solution without (labeled L) and with a permit market (LP). Under the former, countries are assigned abatement burdens ${a}_i^L$ such that these shares sum up to unity:

17 $$ {a}_i^L=\frac{A_i^L}{A^L}\kern0.5em ;{\sum}_i{a}_i^L=1 $$

Given a country’s assigned abatement share, to arrive at the LE, each country chooses the same global abatement level *A* ^*L*^. Each country maximizes

18 $$ L\left({y}_i^c,{A}^L\right)={P}_i{u}_i\left({y}_i^c,{A}^L\right)+{\lambda}_i\left[{R}_i-{P}_i{y}_i^c-{C}_i\left({a}_i^L{A}^L\right)\right] $$

with respect to per capita income and total abatement, giving

19a $$ {u}_{y_i^c}={\lambda}_i $$

19b $$ {P}_i{u}_A={\lambda}_i{C}_{A_i}{a}_i^L $$

Combining both gives

19c $$ {P}_i{u}_A={u}_{y_i^c}{C}_{A_i}{a}_i^L\Rightarrow {P}_i\frac{u_A}{u_{y_i^c}}={a}_i^L{C}_{A_i} $$

Taking summations on both sides of Eq. 19c results in the global sum of marginal benefits to be equal to the weighted sum of marginal cost of abatement:

19d $$ {\sum}_{\kern0.28em i=1}^{\kern0.28em n}{P}_i\frac{u_A}{u_{y_i^c}}={\sum}_i{a}_i^L{C}_{A_i} $$

which is akin to the Samuelson condition. Note that without a permit market, marginal cost of abatement may differ between countries.

The Lindahl planner will assign abatement shares to countries, taking into account that each one maximizes Eq. (18), in such a way that given their assigned abatement shares, they will choose the same global abatement level.^13^ In the empirical section, we show that there is a global abatement level compatible with the constraint that the sum of the abatement shares is unity and that given these shares, each country will choose that level of global abatement as being optimal. However, there is a production inefficiency due to the differentiated, country-specific, marginal cost of abatement, which can be solved by implementing a global permit market.

### Lindahl solution with permit market

A planner imposing the Lindahl burden rule will ensure that for each country, the willingness to pay equals the marginal burden of abatement. A Lindahl planner, also equipped with the power to install a global permit market (LP), will assign target abatement shares ${t}_i^L$, and given these target shares, countries choose the same global abatement level *A* ^*L*^. Given a country’s assigned target abatement *t*_*i*_ *A* ^*L*^ = *T*_*i*_, each country maximizes

20 $$ L\left({y}_i^c,{A}_i,{A}^L\right)={P}_i{u}_i\left({y}_i^c,{A}^L\right)+{\lambda}_i\left[{R}_i-{P}_i{y}_i^c-{C}_i\left({A}_i\right)-q\left({t}_i{A}^L-{A}_i\right)\right] $$

with respect to consumption, domestic abatement, and global abatement, giving

21a $$ {u}_{y_i^c}={\lambda}_i $$

21b $$ \frac{\partial L\left({y}_i^c,{A}_i,{A}^L\right)}{\partial {A}_i}=-{\lambda}_i\left[{C}_{A_i}-q\right]=0 $$

21c $$ \frac{\partial L\left({y}_i^c,{A}_i,{A}^L\right)}{\partial {A}^L}={P}_i{u}_A-{\lambda}_i\left[{C}_{A_i}\frac{\partial {A}_i}{\partial {A}^L}+\frac{\partial q}{\partial {A}^L}\left({t}_i{A}^L-{A}_i\right)+q\left({t}_i-\frac{\partial {A}_i}{\partial {A}^L}\right)\right]=0 $$

Substitution of the first and second into the third first-order condition and rearranging gives

22 $$ {P}_i\frac{u_A}{u_{y_i^c}}=\frac{\partial {A}_i}{\partial {A}^L}\left({C}_{A_i}-q\right)+q\;{t}_i+\frac{\partial q}{\partial {A}^L}\left({t}_i{A}^L-{A}_i\right) $$

which is similar to Eq. 7 above. Due to the permit market, the marginal cost of abatement will never be higher than the permit price, so the first term on the RHS is zero. Therefore, for each country, the marginal rate of substitution between consumption and abatement (the LHS of Eq. 22) is proportional to its target “cost” share (*q t*_*i*_) plus the price effect of a change in the global abatement level. Taking sums on both sides gives

23 $$ {\sum}_{i=1}^n{P}_i\frac{u_A}{u_{y_i^c}}=q{\sum}_{i=1}^n{t}_i+\frac{\partial q}{\partial {A}^L}{\sum}_{i=1}^n\left({t}_i{A}^L-{A}_i\right) $$

The last summation term is zero if the permit market clears, so the Lindahl solution is efficient if the sum of the target shares sum to unity, in which case the population-weighted sum of the marginal rates of substitution between abatement and consumption is equal to the permit price.

Summarizing, the Lindahl solution aligns each country’s willingness to pay and optimal global abatement by adjusting burdens *t*_*i*_, but the price to be paid is that it disregards any reference to polluter pays or ability to pay considerations. To address the question of fairness in terms of contributions of rich and poor, we need to look at the optimum condition for each country, given by Eq. 22. Given that under a permit market the first term is zero and that for countries where actual abatement is close to the assigned target abatement level, the last term will be small and therefore only be of secondary importance, Eq. 22 can approximately be written as

22’ $$ {P}_i{u}_A\approx {t}_iq\kern0.28em {u}_{y_i^c}\kern2em \Rightarrow {MSB}_i^L\approx {MC}_{T_i}^L{MU}_{y_i^C}^L $$

The lower per capita income ${y}_i^c$ is, the higher marginal utility of income ${u}_{y_i^c}$ is; so all other things equal, the lower the marginal cost share *t*_*i*_ *q* is and given the global permit price, the lower the assigned target abatement share ${t}_i={T}_i^L/{A}^L$ is. Therefore, poor countries have to abate little, which is considered as fair according to the ability to pay principle. Now consider a country with a high marginal social benefit of abatement (e.g., located at sea level), as given by the LHS of Eq. 22. The higher it is, the higher the assigned target share is, again given the permit price and per capita income, so countries more affected or concerned with climate change have to abate more, all other things equal. This is reminiscent of the problem of the LE that all countries want to hide their true preferences with respect to abatement if assigned shares are proportional to marginal willingness to pay. This problem however will not arise if the marginal willingness to pay for abatement can be assessed on an objective basis at the country level.^14^ In so far as expected damages from climate change are unrelated to (historical) emissions, the Lindahl solution is violating the polluter pays principle.

In general, with heterogeneous preferences, countries that stand to gain from global warming, so *u*_*A*_ < 0, are entitled to a compensation, e.g., arable land benefits forgone (e.g., in Canada or Russia) if climate change is contained and will have a negative target share. Countries particularly vulnerable to climate change (e.g., the Netherlands, Bangladesh, and islands in the Pacific Ocean all threatened by a sea level rise) will be assigned a higher target contribution because of their higher values of *u*_*A*_. These country characteristics, which determine the country-specific function *u*_*A*_, should be assessed on an objective basis to avoid strategic manipulation of assigned burdens as far as possible. Taking stock, the Lindahl solution enables the IPCC to secure the achievement of the overall abatement objective while at the same time to assess whether the contribution of each country is in line with its willingness to pay for the global public good of limiting global warming.

## Simulation results

In this section, we compare the outcomes of different regimes of burden sharing rules, especially with respect to the extent that optimal global abatement levels are achieved and to the amount of transfer payments relative to the total cost of abatement. For each regime, we measure the level of abatement relative to the optimal abatement level under lump sum (LS; see Appendix 1). The operationalization of the equilibrium conditions for each regime are explained in Appendix 2. We measure redistribution by the share of the transfer payments made by countries with a higher target abatement level than their actual abatement in global abatement costs. It is measured as

24a $$ \mathrm{TP}=\frac{q{\sum}_{i=1,{T}_i>{A}_i}^n\left({T}_i-{A}_i\right)}{\sum_{i=1}^n{C}_i\left({A}_i\right)} $$

TP is an indicator of the share of global abatement costs financed by other countries’ payments on the permit market. For regime LS, in which per capita incomes across countries are equalized and where it does not matter who pays how much, we set TP equal to unity to express that all costs of abatement are shared. If there is no permit market and every country finances its own abatement, the indicator TP is zero by definition.

We also calculate which part of the total cost of abatement is shouldered by the rich countries^15^ (e.g., Annex I, or Europe, Oceania, and North America in case of the five regions; see below), measured as

24b $$ \mathrm{TC}=\frac{\sum_{i=1,\mathrm{rich}}^n\left[q\;\left({T}_i-{A}_i\right)+{C}_i\left({A}_i\right)\right]}{\sum_{i=1}^n{C}_i\left({A}_i\right)} $$

Compared to TP, the measure TC also includes actual abatement costs. If there is no permit market, then it simply measures the abatement cost shares.

We have chosen the parameter *α* for the relative importance of abatement relative to consumption and the cost parameter *c* (see Appendix 2) so that the simulation results simultaneously yield plausible marginal abatement cost (in the range of $20 to $80 per tonne CO_2_), total abatement cost as a share of GDP, and total abatement efforts (e.g., the Stern Review (Stern 2006) recommends a significant reduction of 60–80% by the rich countries in 2050 relative to 1990).^16^ All data are for 2014 and obtained from CAIT Climate Data Explorer (Washington DC, World Resources Institute 2016) and from EDGAR (2009, European Commission Joint Research Centre, Netherlands Environmental Assessment Agency). Table 1 gives an overview of the scores on salient variables if the world is divided into only two blocks, Annex I and Annex II. The Annex I countries comprise the regions Europe and Oceania (EU) and North America (NA). The non-Annex I countries comprise the regions sub-Saharan Africa and Middle East and North Africa (AF), South America and Central America and Caribbean (SA), and Asia (AS). In Table 2, the results are presented if the world is divided into five regions.

**Table 1.**
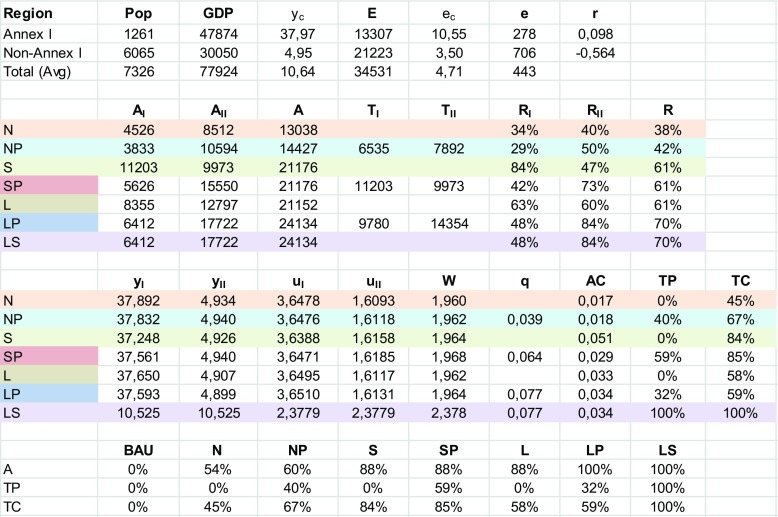
Annex I and II

**Table 2.**
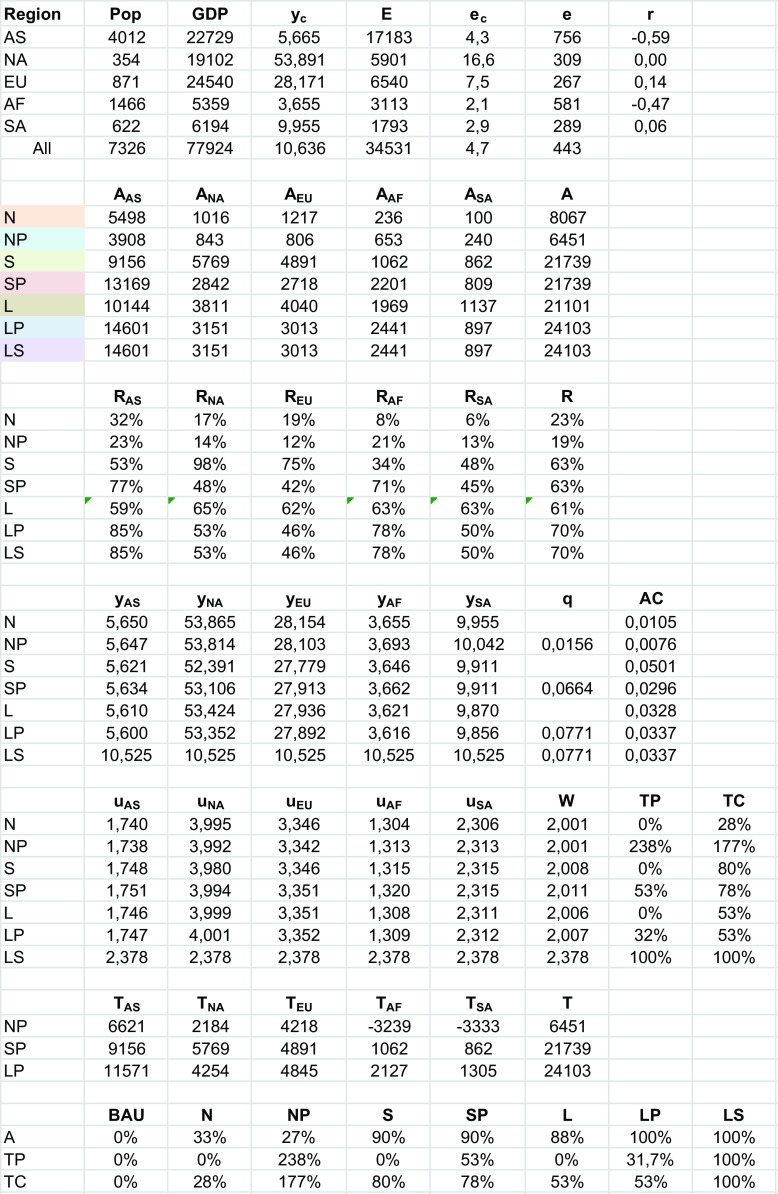
Five regions (Asia = AS, North America = NA, Europe and Oceania = EU, Africa and Middle East = AF, Central and South America = SA)

The top panel in Table 1 contains the descriptive statistics: population (in millions), GDP (in billions USD), income per capita, emissions (in Mt.), emission per capita (in tonnes), emission intensity, and the marginal cost adjustment parameter *r* (see Appendix 2, Eq. 26). Income per capita in Annex I countries is more than seven times as high as in Annex II countries and emissions per capita is more than three times as high. In the second panel, (target) levels of abatement and reduction rates are given, where we take the actual emission levels in 2014 as the business-as-usual outcome (which implies that the Nash outcome is the one where Annex I and I operate as blocks). Firstly, total abatement with a permit market under the same regime is always higher or equal (total abatement under S and SP are set equal by assumption) than without. Secondly, in the shift from N to NP, the Annex II target abatement level under Nash with a permit market is even below its actual abatement without a permit market. This result can be explained by polarization, also described by Cramton and Stoft (2010, p. 6), where the rich will choose an even higher target level because abatement can be bought more cheaply under a permit market, while the poor region will choose an even lower target level of abatement to benefit from permit trading. Thirdly, total abatement under the social planner, without (S) or with (SP) a permit market, is higher than under Lindahl (L). This is because assigning abatement burdens has a dual role for the social planner SP, not only to mitigate global warming but also to redistribute income or welfare. In case of S, Annex I is assigned a very high abatement burden (a reduction rate of 84%, against only 47% for Annex II), because the welfare cost of abatement for the rich countries are relatively small, while the benefits of abatement are global.

The third panel gives information about per capita incomes (y), utility (u), world welfare (W), the permit price (q), average abatement cost (AC), the share of total abatement cost paid for by transfers (TP), and the share in total cost of buyers on the permit market (TC). Not surprisingly, global per capita welfare is at maximum in the lump sum case, but it would not be acceptable for Annex I. Departing from regime N, Annex I would even not be in favor to move to regime NP (due to the polarization effect), nor to S or SP. The only transitions that increase utility for Annex I are the Lindahl regimes L and LP. For Annex II, all other regimes than N are better in utility terms, where S and SP are preferred to L and LP. Taken together, departing from N, only L and LP are Pareto improvements and LP Pareto dominates L, so LP would be a viable outcome.^17^ The equilibrium permit price under LP is 0.077 billion per Mt. CO_2_, which corresponds to $77 per tonne, while average abatement cost per tonne is $34 (due to increasing marginal costs of abatement, average cost is below marginal cost).

**Fig. 1:**
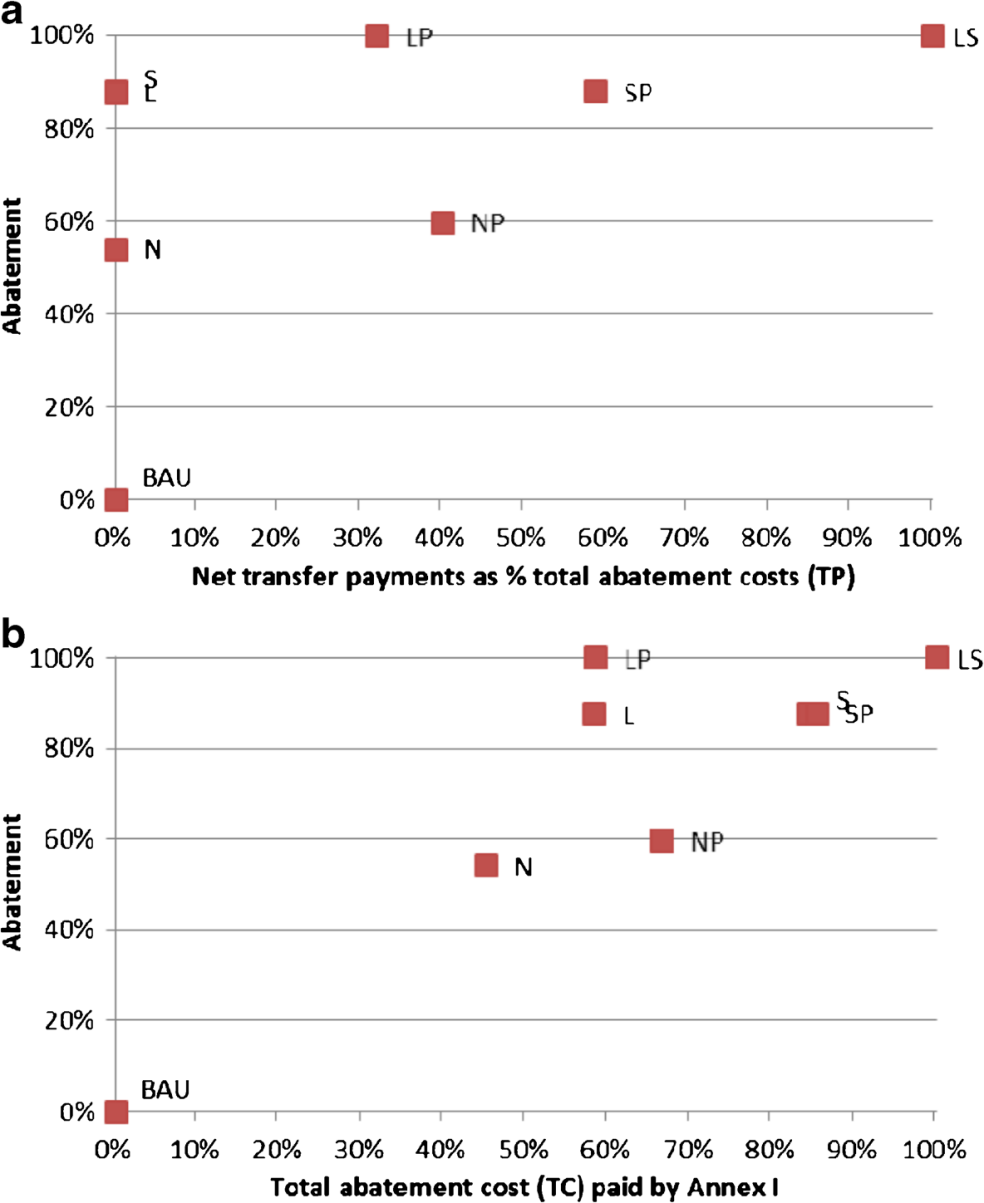
**a** Abatement versus net transfer payments. **b** Abatement versus total abatement cost, Annex I

The last panel gives total abatement relative to (optimal) abatement (A), the share of total abatement cost financed by permits (TP), and the share of the total cost of abatement taken care of by the rich countries (TC). The first two of these measures are illustrated in Fig. 1a, b. Apart from LS, there are four regimes that deliver abatement equal or close to the optimal level under LS. Production efficiency requires the regimes with permit markets. Among the permit market choice set {NP, SP, LP}, LP combines that abatement is at the optimal level and transfer payments as a share of total costs are at minimum. Taking stock, LP (L) are the only regimes that are Pareto-superior to N and combining (near) optimal abatement levels with modest (zero) redistribution payments.

In Table 2, the world is divided in five blocks. CAIT standardly provides a division into the eight geographical regions, Asia (AS), Europe (EUR), Middle East and North Africa (ME), sub-Saharan Africa (AFR), North America (NA), Central America and Caribbean (CAM), South America (SAM), and Oceania (OC). In Table 2, ME and AFR are merged into AF, CAM, and SAM into SA and OC and EUR into EU. In this division, two are rich (North America and Europe with Oceania) and two are poor (Asia and Africa), with Central and South America in between.

**Fig. 2:**
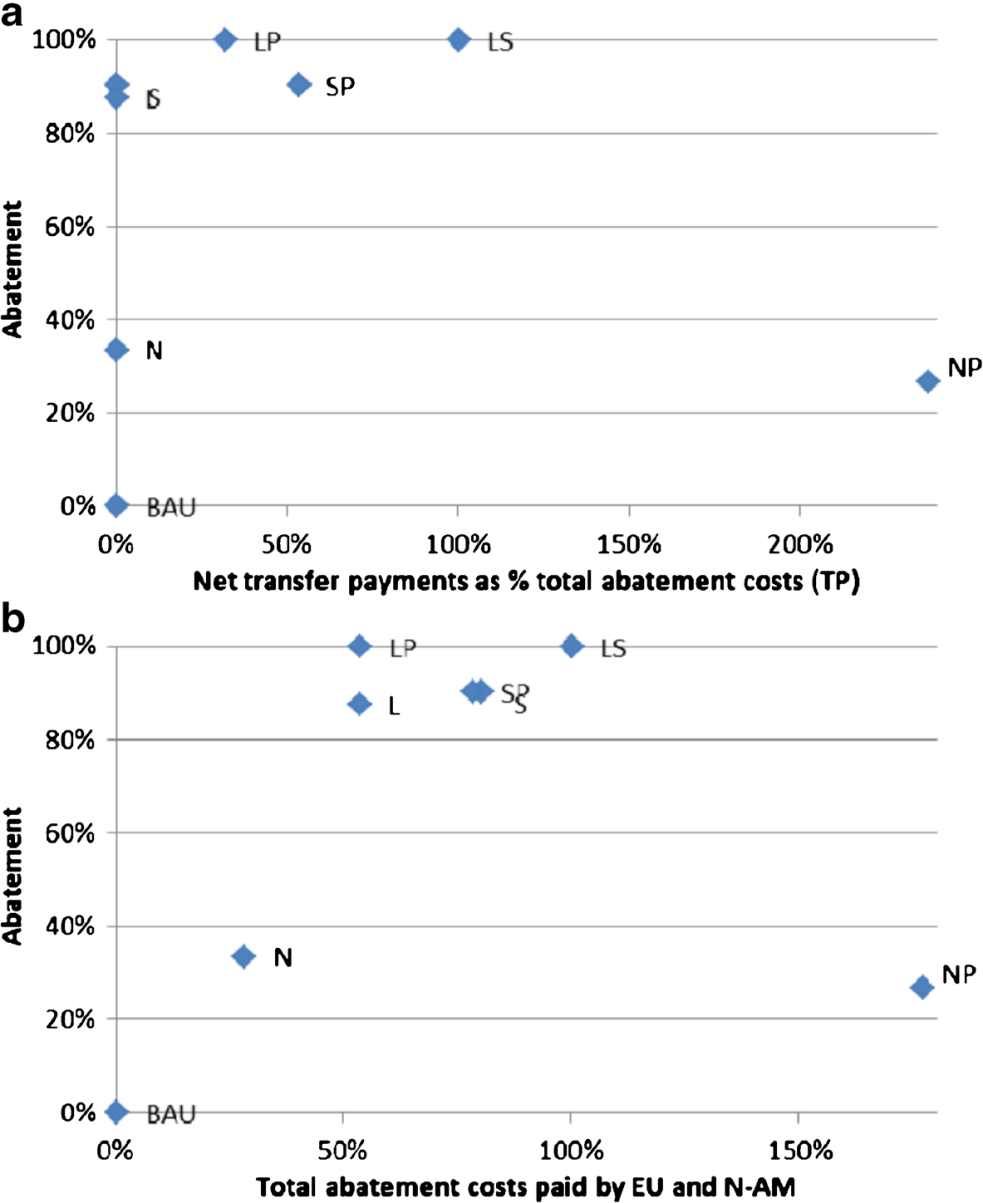
**a** Abatement versus net transfer payments. **b** Abatement versus total abatement cost, EU and North America

Because there are now more players, total abatement levels under Nash are almost halved compared to when Annex I and II operate as blocks. Departing from N, again only L and LP are Pareto improvements for all regions and LP Pareto dominates L. A striking outcome in Table 2 is that for both Africa and South America, it is optimal to choose negative target abatement levels. The extreme polarization leads here to the situation that under NP total abatement is even lower than under N, despite the efficiency gains of a permit market. Note that under regime NP, each player is free to choose its optimal target level. Net revenues from the permit market equals *q*(*A*_*i*_ − *T*_*i*_), so although South America abates 240 Mt., by choosing a target level of − 3333 Mt. and selling permits for the equilibrium price of $15.6 per tonne, it receives $55.7 billion on the permit market, while Africa receives $60.6 billion.^18^ Asia (due to its high population), North American, and Europe and Oceania (due to their high per capita incomes) together pay in total 116.3 billion (their combined target abatement levels of 13,022 Mt. minus their combined actual abatement of 5558 Mt., times $15.6 per tonne), whereas total abatement cost is only 49 billion (total abatement under NP equal to 6451 Mt. times average cost of $7.6 per tonne). The main reason for the outlier position of regime NP in Fig. 2a, b is that transfer payments under NP outweigh total abatement cost, which is not so much due that the average cost of abatement is below the permit price or marginal cost, but because of polarization.

Not surprisingly, total reduction levels (*R*) for the other regimes are almost equal compared to under Annex I and II. Apart from the location of N, shifting downwards and NP, shifting downwards and to the right, Fig. 2a, b therefore gives the same configuration of regimes as in Fig. 1a, b, where regime LP combines optimal abatement with modest transfer payments as a percentage of total cost.^19^

## Summary and conclusions

The Paris agreement of 2015 entails that both developed and developing countries limit their emissions, with 5-yearly reviews to ensure that national contributions are in line with the overall goal to reach at maximum 2 °C global warming, with an aspiration of 1.5 °C. In this paper, the abatement burden sharing rules emerging under different regimes, with and without permit markets, were derived. The sub-optimal level under Nash without permit market is due to both production inefficiencies in abatement and not taking global externalities of national abatement into account. The first sub-optimality can be removed by installing a permit market, but the second requires cooperation or coordination between countries. A social planner without the power to redistribute and without a permit market will impose the rule that the product of the marginal cost of abatement and the marginal utility of income be equalized across countries. This implies high abatement burdens and (marginal) costs for rich and low burdens and costs for poor countries, with the overall abatement level close to optimal. The same social planner but equipped with a permit market can organize transfer payments from rich to poor by assigning high target levels to rich and low target levels to poor regions. The burden sharing rule then has a secondary role of redistribution and without any constraint the social planner’s outcome will be the same as under an omnipotent social planner with lump sum redistribution.

In the simulations, we showed that the transition to a permit market under Nash can lead to polarization, eventually leading to lower overall abatement as shown for the world divided into five regions. Although poor countries prefer the social planner regime with permits the most, only the Lindahl regimes Pareto dominate the Nash regimes, with or without a permit market. Moreover, of all permit regimes, the Lindahl permit market entails the lowest degree of redistribution in the form of net transfer payments from rich to poor. Our preferred burden sharing rule can be summarized as that every country or region shares in the burden to combat climate change in proportion to its benefits, which are determined by the expected damages and adaptation costs. The burden sharing rule implied by the Lindhahl solution therefore provides a criterion to assess the national contributions to curb climate change. The Lindahl solution simultaneously achieves an optimal global abatement level and that each country is expected to contribute according to its willingness to pay, but the price to be paid is to disregard competing principles such as ability to pay and the polluter has to pay. In our analysis, we only differentiated countries by their income per capita and population size. Avenues for further research are to relate the Lindahl solution and the corresponding abatement burdens to country- or region-specific expected damages and associated adaptation costs from climate change as proxies for its willingness to pay (e.g., using an integrated assessment model such as RICE) and a more systematic comparison how the Lindahl solution fares compared to other fairness principles governing burden sharing rules for global public goods.

## Appendix 1: A social planner with lump sum transfers

A social planner with the instrument of lump sum redistribution (labeled LS) maximizes

$$ L\left({y}_i^c,{A}_i,A\right)={\sum}_{i=1}^n{P}_i{u}_i\left({y}_i^c,A\right)+\mu \left[{\sum}_i{A}_i-A\right]+\lambda {\sum}_i\left[{R}_i-{P}_i{y}_i^c-{C}_i\left({A}_i\right)\right] $$

The first term is a summation of welfare over all countries and because of the possibility of lump sum transfers, there is only one world resource constraint given by the last term. Differentiating with respect to ${y}_i^c$, *A*_*i*_, and *A*, respectively,

$$ {\displaystyle \begin{array}{l}{u}_{y_i^c}=\lambda \kern1.00em \\ {}\mu =\lambda {C}_{A_i}\kern1.00em \\ {}{\sum}_i{P}_i{u}_A=\mu \kern1.00em \\ {}\Rightarrow {\sum}_i{P}_i{u}_A=\lambda {C}_{A_i}={u}_{y_i^c}{C}_{A_i}\Rightarrow {\sum}_i{P}_i\frac{u_A}{u_{y_i^c}}={C}_{A_i}\kern0.5em \Rightarrow \frac{MS{B}_w}{M{U}_y}={MC}_A^{LS}\kern1.00em \end{array}} $$

Lump sum redistribution between countries leads to the result that marginal utility of per capita income is equalized across the world (${u}_{y_i^c}=\lambda$), which can only occur if per capita income is equal everywhere. Comparing this result with Eq. 2c, we find that the only difference is that in Eq. 2c, we have in the numerator *MSB*_*i*_ (so the marginal social benefit of abatement only in country *i*) and here *MSB*_*w*_ (marginal social benefit of abatement worldwide). Thus, the marginal cost of abatement ${MC}_A^{LS}$ under a lump sum redistribution scheme exercise is much higher (and therefore the abatement level, assuming increasing marginal cost of abatement) than ${C}_{A_i}^N$ under Nash behavior.

The Lagrangian can be modified to allow for a global permit market, with the additional restriction ∑_*i*_ *A*_*i*_ = ∑_*i*_ *T*_*i*_:

$$ L\left({y}_i^c,{A}_i,A,{T}_i\right)={\sum}_{\kern0.28em i=1}^{\kern0.28em n}{P}_i{u}_i\left({y}_i^c,A\right)+\mu \left[{\sum}_i{T}_i-A\right]-\lambda {\sum}_i\left[{R}_i-{P}_i{y}_i^c-{C}_i\left({A}_i\right)-q\left({T}_i-{A}_i\right)\right] $$

which yields

$$ {\displaystyle \begin{array}{l}{u}_{y_i^c}=\lambda \kern1.00em \\ {}\mu =\lambda \left({C}_{A_i}-q\right)\Rightarrow {C}_{A_i}=q\kern1.00em \\ {}{\sum}_i{P}_i{u}_A=\mu \kern1.00em \\ {}\mu =\lambda \kern0.1em q\kern1.00em \\ {}\Rightarrow {\sum}_i{P}_i{u}_A=\lambda \kern0.28em q={u}_{y_i^c}{C}_{A_i}\Rightarrow {\sum}_i{P}_i\frac{u_A}{u_{y_i^c}}={C}_{A_i}=q\kern0.5em \Rightarrow \frac{MS{B}_w}{M{U}_y}={MC}_A^{LSP}=q\kern1.00em \end{array}} $$

So, the Samuelson condition is met; there is a uniform marginal cost of abatement and marginal utility of income for consumption is equalized, which also applies without a permit market.

## Appendix 2: Empirical specification

Following Nordhaus (1991), Bohm and Larsen (1994), Eyckmans et al. (1993), and Okada (2007), we define the marginal cost function of abatement as

25 $$ {MC}_{\mathrm{USA}}=-c\;\ln \left(1-{A}_{\mathrm{USA}}/{E}_{\mathrm{USA}}\right) $$

so marginal cost for the USA are an increasing function of its emission reduction rate *A*/*E*.^20^

Other countries or parts of the world may have carbon intensities (*e* = *E*/*Y*) higher or lower than the USA. If it is lower, e.g., because in the past already abatement measures were adopted, it is at the margin costlier to reduce emissions further. For a country or region with a carbon intensity different from that in the USA, the marginal cost function relative to the USA is

26 $$ {\displaystyle \begin{array}{llll}{MC}_i=-c\ln \left(1-\frac{A_i/{E}_i}{1-{r}_i}\right)\hfill & {r}_i=1-\frac{e_i}{e_{\mathrm{USA}}}\hfill & \mathrm{if}\hfill & {e}_i<{e}_{\mathrm{USA}}\hfill \\ {}\hfill & {r}_i=\frac{e_{\mathrm{USA}}}{e_i}-1\hfill & \mathrm{if}\hfill & {e}_i>{e}_{\mathrm{USA}}\hfill \end{array}} $$

The carbon intensity for the USA in 2014 was 308 t per million US$, which amounts to 0.3 kg carbon dioxide per US$ production. For Annex I as a whole, it was 278, and for Annex II, it was 706, resulting in *r*_I_ = 0.098 and *r*_II_ = −0.564 (note that *r*_USA_ is equal to zero).

For the simulation, we assume a quasi-linear utility function of the form

27 $$ {u}_i\left({y}_i^c,A\right)=\ln \left({y}_i^c\right)+{\alpha}_iA\kern2em $$

where the parameter *α*_*i*_ denotes the importance of the quality of the atmosphere, measured by global abatement, relative to per capita income for consumption. Differentiation of Eq. 27 gives that the marginal utility of income ${u}_{y_i^c}$ equals $1/{y}_i^c$ and marginal utility of abatement *u*_*i* , *A*_ equals *α*_*i*_. In what follows, we will highlight the most important equilibrium conditions derived in the previous sections together with the constraints to solve the models.

The most easy model to solve is the lump sum social planner (see Appendix 1), equalizing per capita incomes for consumption (${y}_w^c$),

28 $$ {\sum}_{i=1}^n{P}_i\frac{u_A}{u_{y_i^c}}={q}^{LS}\Rightarrow \alpha {P}_w{y}_w^c={q}^{LS}\Rightarrow {q}^{LS}=\alpha {Y}_w $$

and using Eq. 26 abatement levels can be derived from

29 $$ {MC}_i={q}^{LS}=-c\ln \left[1-\frac{A_i^{LS}}{E_i\left(1-{r}_i\right)}\right]\Rightarrow {A}_i^{LS}={E}_i\left(1-{r}_i\right)\left(1-\exp \left(-{q}^{LS}/c\right)\right) $$

For the model of Nash without a permit market (denoted by superscript *N*), the condition to be met is Eq. 2c, which in combination with Eqs. 25–27 gives

30 $$ {P}_i\frac{u_{i,A}}{u_{y_i^c}}={MC}_{A_i}^N\Rightarrow {\alpha}_i{Y}_i=-c\ln \left[1-\frac{A_i^N}{E_i\left(1-{r}_i\right)}\right]\Rightarrow {A}_i^N={E}_i\left(1-{r}_i\right)\left(1-\exp \left(-{\alpha}_i{Y}_i/c\right)\right) $$

using that ${P}_i{y}_i^c\equiv {Y}_i$. Total cost of abatement can be derived from integrating the marginal cost function of abatement (see also Okada 2007, p. 245; Bohm and Larsen 1994, p. 229):

31 $$ {C}_i\left({A}_i\right)={\int}_0^{A_i}-c\ln \left[1-\frac{x}{E_i\left(1-{r}_i\right)}\right] dx=c\left\{\left({E}_i\left(1-{r}_i\right)-{A}_i\right)\ln \left[1-\frac{A_i}{E_i\left(1-{r}_i\right)}\right]+{A}_i\right\} $$

Substituting the abatement cost function and the abatement level derived from the equilibrium condition of Eq. 30 in the resource constraint ${R}_i={P}_i{y}_i^c+{C}_i\left({A}_i\right)$ gives an expression which can be numerically solved for the only unknown, consumption.

For the Nash outcome with a global permit market (denoted by superscript *NP*), the permit price is not dependent on the permit allocation across countries, but only on the relative importance of climate quality versus income, for simplicity assumed to be uniform (so *α*_*i*_ = *α*), and the number of countries. Using Eq. 8, it follows that

8 $$ {\sum}_{i=1}^n{P}_i\frac{u_A}{u_{y_i^c}}={nq}^{NP}\Rightarrow \alpha {Y}_w={nq}^{NP} $$

Because of the permit market, marginal costs are uniform:

32 $$ {MC}_i^{NP}={q}^{NP}=-c\ln \left[1-\frac{A_i^{NP}}{E_i\left(1-{r}_i\right)}\right] $$

So for each country, the optimal abatement level^21^ and abatement cost become

33 $$ {A}_i^{NP}={E}_i\left(1-{r}_i\right)\left(1-\exp \left(-{q}^{NP}/c\right)\right) $$

34 $$ {C}_i^{NP}\left({A}_i^{NP}\right)=q\;\left({A}_i^{NP}-{E}_i\left(1-{r}_i\right)\right)+c\;{A}_i^{NP} $$

Aggregating all country abatements stated by Eq. 33,

35 $$ {A}^{NP}={\sum}_{i=1}^n{A}_i^{NP}=\left(1-\exp \left(-{q}^{NP}/c\right)\right){\sum}_{i=1}^n\left(1-{r}_i\right){E}_i\Rightarrow {q}^{NP}=-c\ln \left[1-{A}^{NP}/{\sum}_{i=1}^n\left(1-{r}_i\right){E}_i\right] $$

which indeed shows that the equilibrium permit price is only dependent on the global abatement level. Taking the derivative of Eq. 35 and because a clearing permit market implies *A* = *T*:

36 $$ {q}_T^{NP}={q}_A^{NP}=\frac{c\;}{\sum_{i=1}^n\left(1-{r}_i\right){E}_i-{A}^{NP}} $$

Rewriting the expression for *q* ^*NP*^ in Eq. 35 as ${A}^{NP}=\left[1-\exp \left(-{q}^{NP}/c\right)\right]{\sum}_{i=1}^n\left(1-{r}_i\right){E}_i$ and substituting in Eq. 36 gives

36’ $$ {q}_A^{NP}=\frac{c\;\exp \left({q}^{NP}/c\right)}{\sum_{i=1}^n\left(1-{r}_i\right){E}_i} $$

Finally, using Eq. 7,

7 $$ {P}_i\frac{u_A}{u_{y_i^c}}={q}^{NP}+{q}_T\left({T}_i^{NP}-{A}_i^{NP}\right)\Rightarrow {T}_i^{NP}={A}_i^{NP}+\frac{\alpha {Y}_i-{q}^{NP}}{q_T^{NP}} $$

Turning to the social planner without permit market (denoted by superscript S), the equilibrium condition Eq. 11 translates into

37 $$ \frac{\sum_{\kern0.28em j=1}^{\kern0.28em n}{P}_j{u}_A^j}{u_{y_i^c}}={MC}_i^S\Rightarrow \alpha {P}_w{y}_i^c=-c\ln \left[1-\frac{A_i^S}{E_i\left(1-{r}_i\right)}\right]\Rightarrow {A}_i^S={E}_i\left(1-{r}_i\right)\left(1-\exp \left(-\alpha {P}_w{y}_i^c/c\right)\right) $$

The system of equations for the social planner with a permit market is easy to solve due to the decision to pitch the target-level abatements at the actual abatement levels if there would be no permit market, so ${T}_i^{SP}={A}_i^S$. Doing so ensures that no country will object to install the permit market, because it will never be more expensive under the permit market to meet the same commitment as without a permit market. Using that marginal cost will be equal to the permit price, as stated by Eq. 13b, each country’s abatement is given by Eq. 33, except for *q* ^*SP*^ instead of *q* ^*NP*^. Taking the sum on both sides and solving for *q* ^*SP*^ gives the same expression as the RHS of Eq. 33 and differentiation to *q* ^*SP*^ gives the same as Eq. 36.

For the Lindahl solution without a permit market, Eq. 19bb can be expressed as

38 $$ {P}_i{u}_A={\lambda}_i{C}_{A_i}{a}_i^L\Rightarrow \alpha {P}_i{y}_i^c=-{a}_i^Lc\ln \left[1-\frac{a_i^L{A}^L}{E_i\left(1-{r}_i\right)}\right] $$

The abatement costs as given by Eq. 31 becomes

39 $$ {C}_i\left({A}_i\right)=c\left\{\left({E}_i\left(1-{r}_i\right)-{a}_i^L{A}^L\right)\ln \left[1-\frac{a_i^L{A}^L}{E_i\left(1-{r}_i\right)}\right]+{a}_i^L{A}^L\right\} $$

Substituting *C*_*i*_(*A*_*i*_) into the budget constraint ${y}_i^c=\left({R}_i-{C}_i\left({A}_i\right)\right)/{P}_i$ and subsequently ${y}_i^c$ into Eq. (38) gives an expression that can numerically be solved for any global abatement level. The chosen Lindahl solution is that level of global abatement for which the sum of abatement shares sum to unity.

Finally, for the Lindahl solution with a permit market, ${\sum}_{i=1}^n{t}_i=1$ and ${\sum}_{i=1}^n{A}_i^{LP}={A}^{LP}$. For each country, it is optimal to abate up to the point where the cost will be equal to the permit price, so

40a $$ {MC}_i={q}^{LP}=-c\ln \left[1-\frac{A_i^{LP}}{E_i\left(1-{r}_i\right)}\right]\Rightarrow {A}_i^{LP}={E}_i\left(1-{r}_i\right)\left(1-\exp \left(-{q}^{LP}/c\right)\right) $$

Also, for all countries together, it must be the case that

40b $$ {q}^{LP}=-c\ln \left[1-\frac{A^{LP}}{\sum_{i=1}^n\left(1-{r}_i\right){E}_i}\right]\Rightarrow {A}^{LP}={\sum}_{i=1}^n\left(1-{r}_i\right){E}_i\left(1-\exp \left(-{q}^{LP}/c\right)\right) $$

Moreover, each country contributes according to marginal willingness to pay

41 $$ {P}_i\frac{u_A}{u_{y_i^c}}={q}^{LP}\;{t}_i+{q}_A^{LP}\left({t}_i{A}^L-{A}_i\right) $$

with ${q}_A^{LP}$ similar as in Eq. 36. Finally, the budget constraints to be met are

42 $$ {R}_i={P}_i{y}_i^c+{C}_i\left({A}_i\right)+q\left({t}_i{A}^{LP}-{A}_i\right) $$

with cost functions similar to Eq. 34, so ${C}_i^{LP}\left({A}_i^{LP}\right)={q}^{LP}\;\left({A}_i^{LP}-{E}_i\left(1-{r}_i\right)\right)+c\;{A}_i^{LP}$. In the simulation where the world is divided into Annex I and Annex II, there are 11 unknowns $\left\{{A}_I^{LP},{A}_{II}^{LP},{A}^{LP},{t}_I,{t}_{II},{q}^{LP},{q}_A^{LP},{y}_I^c,{y}_{II}^c,{C}_I,{C}_{II}\right\}$ and 11 equations, which can be solved numerically.

## Footnotes

1 See https://unfccc.int/resource/docs/2015/cop21/eng/l09r01.pdf.

2 Giersch (2007, p. 1), comparing Lindahl with the Nash outcome, also mentions that although the Lindahl equilibrium is considered as one of the cornerstones of public finance, it is also “dismissed as unconvincing.”

3 This can be can be modeled as *L* = *u*_*j*_(*y*_*j*_, *A*__*j*_ + *a*_*j*_) + *λ*(*r*_*j*_ − *y*_*j*_ − *c*_*j*_(*a*_*j*_)), where subscript *j* refers to individuals. The optimum condition is ${u}_{j,A}/{u}_{y_j}={c}_{a_j}^N$ with *a*_*j*_ abatement by *j*. In that case, the private marginal rate of substitution between abatement and consumption is equated against private marginal cost of abatement. Compared with the outcome of Eq. 2c, even the positive externalities within one’s own country are ignored.

4 Under the zero Nash conjecture, each country takes the chosen target levels by others as given, so a change in its own target level will lead to an equal change in total abatement.

5 The social optimum would be that the price of the permit equals the LHS of Eq. 8.

6 With lump sum redistribution, ${u}_{y_i^c}=\lambda$, so marginal utility of income per capita is uniform across countries (see Appendix 1).

7 Alternatively, SP may impose a uniform global carbon tax, with the tax equal to the equilibrium permit price.

8 SP will assign target abatement levels such that ${y}_i^c=\left[{R}_i-{C}_i\left({A}_i\right)-q\left({T}_i-{A}_i\right)\right]/{P}_i={y}_w^c$, while at the same time, the global sum of abatement equals the exogenously given or optimal global abatement target, with large negative target levels for poor and large positive target levels for rich countries.

9 On the same footing, Shiell (2003, p. 44) notes that “If negative allocations were permitted for some countries, then the system would be equivalent to unrestricted lump sum transfers.”

10 Elzen and Lucas (2003, Sect. 5) provides an overview of ten different burden rules based on four equity principles, namely, egalitarian (equal caps), sovereignty (grandfathering), polluter pays, and capability (ability to pay).

11 In terms of a two-person household and the cleanliness of the house as the public good, it might be that one member has a higher ability to clean or is more polluting than the other, but at the same time has a much lower preference for a clean house, so a lower willingness to clean.

12 Also, Giersch (2007, p. 18) considers this approach of focusing on abatement rather than cost shares more realistic.

13 In terms of the 2015 Paris agreement, the main achievement is the commitment of all countries to limit global warming to well below 2 °C, which corresponds to a global abatement level. In our approach, in the five yearly global stocktakes, the “nationally determined contributions” have to be compared with the contributions specified by the Lindahl solution to see where they do not come up to the mark.

14 For instance, Tol (2002) provides estimates of the damage of climate change for nine world regions for the period of 2000–2200. Although the negative impacts dominate, for some regions at some intervals, the total impact is positive. Also, the Stern Review (2007, p. 8) states that “In higher-latitude regions, such as Canada, Russia, and Scandinavia, climate change may lead to net benefits for temperature increases of 2 or 3 °C, through higher agricultural yields, lower winter mortality, lower heating requirements, and a possible boost to tourism. But these regions will also experience the most rapid rates of warming, damaging infrastructure, human health, local livelihoods, and biodiversity.”

15 Both indicators overestimate in what they purport to measure, because under the assumption of increasing marginal cost of abatement, the average cost will always be below the permit price.

16 The value for *α* is set equal to 10^−5^. The cost parameter *c* is set equal to 0.101, double the value used by Nordhaus (1991) and Bohm and Larsen (1994). They use a value for *c* of 185.2, but since we express emissions in carbon dioxide, where 1 kg carbon corresponds to 3.67 kg CO_2_, we get 185.2/3.67 = 50.5. Since we measure abatement in Mt. and GDP in billions, we have to divide 50.5 by 1000, and doubling (to adjust for inflation since the early 90s and increasing cost of abatement) gives our chosen value of 0.101. More specifically, given the specifications of the utility function in Eq. 27 and the marginal cost function in Eq. 26 in Appendix 2, the choice of the parameters is such that total cost of abatement will be a small share of GDP, in line with the Stern Review. The Report of the High-Level Commission on Carbon Prices (2017, p. 3) recommends a carbon price in the range of $40–$80 by 2020.

17 Buchholz et al. (2006, p. 33) show that a move from Nash to Lindahl does not necessarily entail a Pareto improvement because of two countervailing effects. First, all countries will gain in terms of benefits derived from overcoming the sub-optimal low Nash provision level of the public good. Second, the move from Nash to Lindahl might however entail higher costs for some (poor) countries, notably if under the Nash outcome they were free-riding on the contributions of others (often manifested in the form of exploitation of the rich by the poor). The second effect may outweigh the first effect, which is more likely the poorer the country is (the more it was easy-riding under Nash) and the higher its marginal preference for abatement (the higher its assigned burden under Lindahl), but as the number of participating countries increase, it becomes more likely that the first effect will be dominant.

18 Hof et al. (2010) argue that these net transfers can be considered as compensation for more severe climate change damages and higher adaptation costs in, for instance, Africa.

19 Although it is possible to further disaggregate the simulation to the individual country level, under the assumptions made the shift from N to LP will be a Pareto improvement because the positive effect of the higher abatement level under Lindahl compared to Nash will be even stronger than when the world is divided into two or five regions.
